# The Combination of Chest Computed Tomography and Standard Electrocardiogram Provides Prognostic Information and Pathophysiological Insights in COVID-19 Pneumonia

**DOI:** 10.3390/jcm10143031

**Published:** 2021-07-07

**Authors:** Matteo Bertini, Emanuele D’Aniello, Alberto Cereda, Marco Toselli, Filippo Maria Verardi, Luca Rossi, Daniela Aschieri, Alberto Monello, Marco Manfrini, Davide Vignale, Anna Palmisano, Antonio Esposito, Roberto Ferrari, Claudio Rapezzi, Francesco Giannini

**Affiliations:** 1 Cardiological Center, Translational Medicine Department, University of Ferrara, 44121 Ferrara, Italy; emanuele.daniello2@gmail.com (E.D.); filippomaria.verardi@gmail.com (F.M.V.); fri@unife.it (R.F.); claudio.rapezzi@unife.it (C.R.); 2 Maria Cecilia Hospital, 48033 Cotignola, Italy; tskcer@hotmail.it (A.C.); marco.toselli2@gmail.com (M.T.); mmanfrini@gvmnet.it (M.M.); giannini_fra@yahoo.it (F.G.); 3 Cardiology Unit, Ospedale Guglielmo da Saliceto, 29121 Piacenza, Italy; dr.luca.rossi.20@gmail.com (L.R.); Alberto.monello@ausl.pc.it (A.M.); 4 Cardiology Unit, Ospedale Civile di Castel San Giovanni, 29015 Castel San Giovanni, Italy; dani.aschieri@gmail.com; 5 Clinical and Experimental Radiology Unit, Experimental Imaging Center, IRCCS San Raffaele Scientific Institute, Ospedale San Raffaele, 20132 Milano, Italy; vignale.davide@hsr.it (D.V.); palmisano.anna@hsr.it (A.P.); esposito.antonio@hsr.it (A.E.); 6 School of Medicine, Università Vita-Salute San Raffaele, 20132 Milano, Italy

**Keywords:** COVID-19, chest CT, ECG

## Abstract

Aims. Several studies have unveiled the great heterogeneity of COVID-19 pneumonia. Identification of the “vascular phenotype” (involving both pulmonary parenchyma and its circulation) has prognostic significance. Our aim was to explore the combined role of chest computed tomography (CT) scan and electrocardiogram (ECG) at hospital admission in predicting short-term prognosis and to draw pathophysiological insights. Methods and Results. We analyzed the chest CT scan and ECG performed at admission in 151 consecutive COVID-19 patients admitted between 20 March and 4 April 2020. All-cause mortality within 30 days was the primary endpoint. Median age was 71 years (IQR: 62–76). Severe pneumonia was present in 25 (17%) patients, and 121 (80%) had abnormal ECG. During a median follow-up of 7 days (IQR: 4–13), 54 (36%) patients died. Deceased patients had more severe pneumonia than survivors did (80% vs. 64%, *p* = 0.044). ECG in deceased patients showed more frequently atrial fibrillation/flutter (17% vs. 6%, *p* = 0.039) and acute right ventricular (RV) strain (35% vs. 10%, *p* < 0.001), suggesting the “vascular phenotype”. ECG signs of acute RV strain (HR 2.46, 95% CIs 1.36–4.45, *p* = 0.0028) were independently associated with all-cause mortality in multivariable analysis, and in the likelihood ratio test, showed incremental prognostic value over chest CT scan, age, and C-reactive protein. Conclusions. Combining chest CT scan and ECG data improves risk stratification in COVID-19 pneumonia by identifying a distinctive phenotype with both parenchymal and vascular damage of the lung. Patients with severe pneumonia at chest CT scan plus ECG signs of acute RV strain have an extremely poor short-term prognosis.

## 1. Introduction

Several recent studies have unveiled the great heterogeneity of COVID-19 pneumonia, ranging from mild, rapidly improving symptoms to a devastating accelerated decline of lung function [1,2,3]. In particular, different phenotypes have been described based on respiratory parameters, the presence of dead space, and coagulation markers [4,5]. Grasselli and coworkers described a subgroup of mechanically ventilated patients with a distinct respiratory phenotype characterized by low static lung compliance, increased dead space, high D-dimer concentrations, and a poor short-term prognosis [6]. Autopsy studies specified that this phenotype involves both the pulmonary parenchyma and its circulation, showing pulmonary vascular endothelialitis, thrombosis, and angiogenesis [7]. Notably, this “vascular phenotype” is not necessarily the result of pneumonia associated with thromboembolic events; more frequently, it is an intrinsic phenomenon of thrombosis in situ. Identification of the vascular phenotype has clinical significance. Although several clinical, laboratory, and imaging variables have been tested as prognosticators [8,9,10,11], few data are available on the use of a simple and widespread test such as standard electrocardiogram (ECG) [12,13,14].

The aim of this study was to explore the combined role of two simple examinations in predicting short-term prognosis in patients with COVID-19 pneumonia—namely, chest computed tomography (CT) scan and ECG performed at admission—and to draw pathophysiological insights on this heterogeneous condition.

## 2. Methods

### 2.1. Study Setting and Patient Cohort

This study is part of the ELCOVID project, a large multicenter project aimed at assessing the standard ECG features of a large cohort of consecutive COVID-19 patients hospitalized within an endemic area of Italy. It was approved by the local Ethics Committee (identifier: 385/2020/Oss/AOUFe) and is registered on ClinicalTrials.gov (identifier: NCT04367129). Four hospitals of the Emilia Romagna region (Arcispedale S. Anna, Ferrara; Ospedale Guglielmo da Saliceto, Piacenza; Ospedale Civile di Castel San Giovanni, Piacenza; and Maria Cecilia Hospital, Cotignola, Ravenna) took part in the study. The aim was to test the combined role of chest CT scan and standard ECG, performed at admission, in predicting the 30-day prognosis of patients with COVID-19-related pneumonia. We analyzed the chest CT scan and ECG performed at hospital admission of consecutive COVID-19 patients admitted between 20 March and 4 April 2020. The inclusion criteria were age > 18 years, COVID-19 confirmed by RNA detection of severe acute respiratory syndrome coronavirus 2 (SARS-CoV-2) at pharyngeal swab, radiologic evidence of definite COVID-19-related pneumonia, and chest CT scan and 12-lead surface ECG performed at hospital admission. CT scan and ECG were both performed on day 1 of hospitalization in all the patients. Patients were excluded if chest CT scan and 12-lead ECG were not both performed on the same day.

### 2.2. Data Collection

Demographic and clinical characteristics, blood tests at hospital admission, and medication during hospitalization were collected from patients’ medical record (Table 1). Cardiovascular disease defined a group of disorders of the heart and blood vessels (coronary heart disease, cerebrovascular disease, and peripheral arterial disease). Admission to the intensive care unit (ICU) and/or need for invasive or noninvasive mechanical ventilation were also recorded.

Chest CT scans were acquired with a standard non-gated chest CT protocol, always on multidetector scanners with at least 16 detector rows. They were reconstructed at each site with sharp kernel and visualized at the core lab using a standard lung window (width 1400 HU; center –450 HU). An expert cardio-thoracic radiologist blinded to patients’ clinical data analyzed the CT scans. For lung parenchyma evaluation, a semi-quantitative pneumonia scoring was applied: no–mild pneumonia (0–50%); moderate–severe pneumonia (51–100%); severe pneumonia (76–100%). Two experienced cardiologists independently performed the ECG analysis according to standard definitions and diagnostic criteria [15]. The ECG parameters collected are reported in Table 2. We considered the S1Q3T3 pattern, in isolation or associated with right bundle branch block (RBBB), or isolated RBBB as ECG signs of acute right ventricular (RV) strain [13,16,17].

### 2.3. Outcome and Prognostic Endpoint

Patients were followed-up for a maximum of 30 days and all-cause mortality within 30 days was the primary endpoint event. From the various demographic, clinical, laboratory, chest CT, and ECG variables recorded, we identified independent predictors of all-cause mortality through univariable and multivariable Cox regression analysis.

### 2.4. Statistical Analysis

Descriptive statistics were performed on the overall population, grouped by exposure (chest CT scan and standard ECG at hospital admission) and outcome (all-cause death). Continuous variables are presented as mean (standard deviation, SD) or median (interquartile range, IQR) and categorical variables as counts and proportions (%). For continuous variables, differences between groups were compared using the *t*-test and one-way analysis of variance or the Wilcoxon rank sum test and Kruskal–Wallis test for parametric and nonparametric data, respectively. Only variables with missing values <10% of the total number of observations were retained for downstream analysis in the analysis dataset. Multivariate imputation by chained equations was performed on these variables to reduce missing data bias. The dataset for continuous variables was cleaned to remove outliers.

From the various demographic, clinical, laboratory, chest CT, and ECG variables recorded, independent predictors of all-cause mortality were identified by univariable and multivariable Cox regression analysis. Cox proportional hazards with robust variance regression modelling was used to analyze the effect of variables on mortality. All baseline variables were tested in a univariate model, and those found to be significant (*p* < 0.05) were included in an adjusted multivariate Cox regression analysis. Results are reported as hazard ratios (HR) with associated 95% confidence intervals (CI). The multicollinearity was examined using the variance inflation factor (VIF), and variables with VIF > 5 were excluded from the model. A model was built including covariates and the two predictors of interest (chest CT scan and standard ECG at hospital admission). The *p*-value related to the likelihood ratio was calculated and the full model was obtained by variable selection performed by a backward stepwise algorithm based on Bayesian information criterion (BIC) minimization. To evaluate the contribution of ECG data at admission over the reduced model without ECG admission data, we used the log-likelihood ratio test along with BIC variation calculation to compare the goodness of fit of the two competing statistical models. To evaluate the performance of the selected model in terms of its discrimination capability for all-cause mortality, we plotted receiver operating characteristic (ROC) curves, calculating the area under the curve (AUC) associated with the models.

## 3. Results

### 3.1. Patient Population

Out of 743 patients screened, 592 were excluded due to ECG not present or performed on the same day as chest CT (*n* = 351), chest CT not present or performed on the same day as ECG (*n* = 119), or COVID-19 not confirmed by pharyngeal swab (*n* = 122). The remaining 151 consecutive patients constituted the study population. Median age was 71 years (IQR 62–76), and 95 (63%) patients were male (Table 1).

### 3.2. Chest CT and ECG Findings

Chest CT scan revealed diffuse involvement of pulmonary parenchyma in 121 (80%) patients affecting all five lobes, and moderate to severe pneumonia was present in 25 (17%) patients.

The ECG was abnormal in 121 (80%) patients, and 15 (10%) patients had atrial fibrillation/flutter. ECG signs of acute RV strain were present in 29 (19%) patients, whereas left anterior hemiblock and left bundle branch block were present in 6 (4%) and 4 (3%) patients, respectively. Non-specific repolarization abnormalities were present in 23% of patients, and the median corrected QT interval was 445 (IQR 414–464) ms.

### 3.3. Prognostic Analysis 

During a median follow-up of 7 (IQR 4–13) days, 54 (36%) patients died (52 of respiratory failure, 2 from cardiogenic shock). The main baseline differences between survivors and deceased patients are reported in Table 1 and Table 2. There were no differences in terms of sex, cardiovascular risk factors, cardiac and extra-cardiac disease, blood pressure, oxygen saturation, or use of vasopressor drugs, but deceased patients were older (74 (IQR 65–79) vs. 69 (IQR 58–65) years, *p* = 0.006). Survivors were more often admitted to ICU (51% vs. 22%, *p* = 0.001) and more frequently underwent mechanical ventilation (45% vs. 19%, *p* = 0.001) compared with those who died. Of note, patients who were admitted to ICU and who received invasive mechanical ventilation were significantly younger than those who were not admitted to ICU (65 (IQR 58–71) vs. 75 (IQR 67–81) years, *p* < 0.001) or did not receive this treatment (67 (IQR 61–71) vs. 74 (IQR 65–80) years, *p* = 0.010).

Regarding blood tests, deceased patients had higher levels of D-dimer (1.48 (IQR 0.90–2.46) vs. 1.00 (IQR 0.67–1.65) mg/L, *p* = 0.008) and C-reactive protein (13.1 (IQR 7.4–18.0) vs. 10.0 (IQR 5.1–14.2) mg/dL, *p* = 0.012). At chest CT scan, deceased patients had more severe pneumonia than survivors (80% vs. 64%, *p* = 0.044). The ECG in deceased patients showed more frequently the presence of atrial fibrillation/flutter (17% vs. 6%, *p* = 0.039) and ECG signs of acute RV strain (35% vs. 10%, *p* <0.001). 

**Figure 1:**
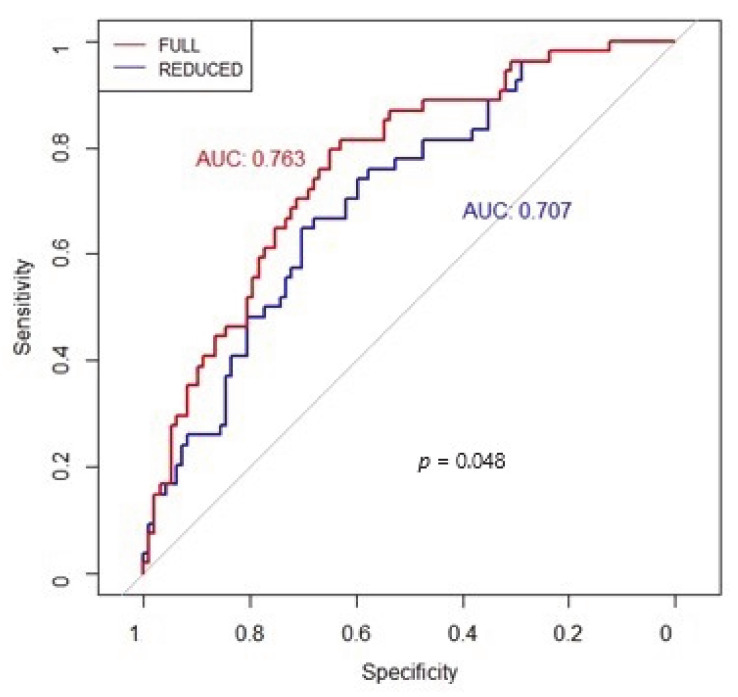
Receiver operating characteristic (ROC) curves were plotted to evaluate the performance of the selected Cox models showing the incremental prognostic value of electrocardiographic (ECG) signs of acute right ventricular (RV) strain (FULL model) over chest computed tomography (CT) scan without ECG signs of acute RV strain (REDUCED model).

**Figure 2:**
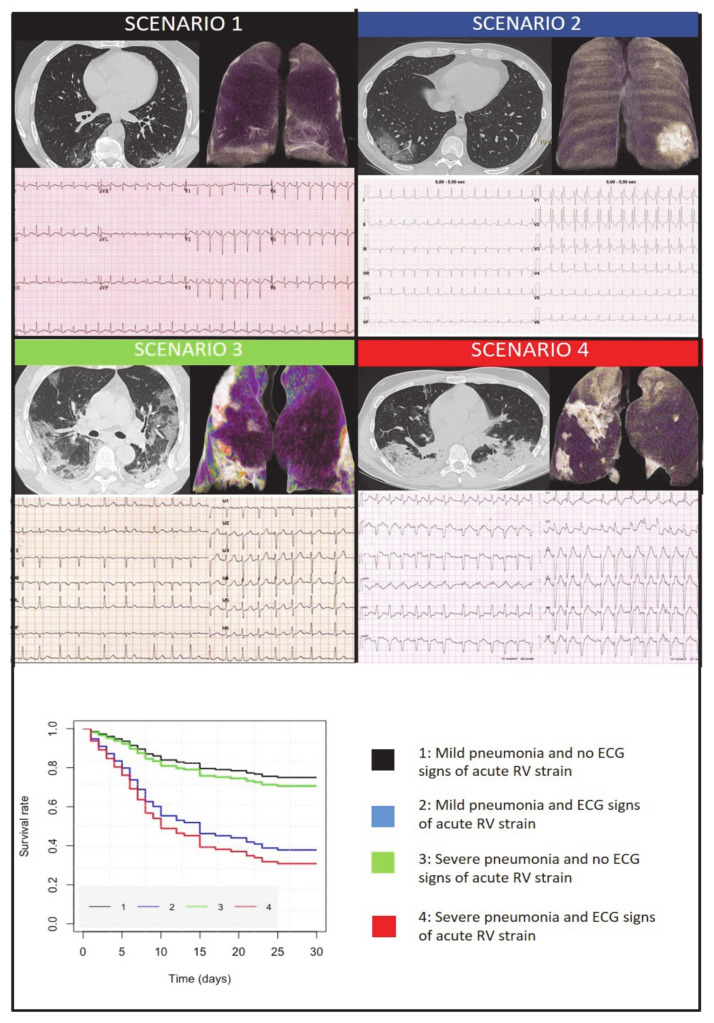
Four clinical scenarios of COVID-19 patients based on combined electrocardiographic (ECG) and chest computed (CT) information (axial image and 3D volumes rendering of lungs in antero-posterior view). **Scenario 1**: mild pneumonia without ECG signs of acute right ventricular (RV) strain; **Scenario 2**: mild pneumonia with ECG signs of acute RV strain; **Scenario 3**: severe pneumonia without ECG signs of acute RV strain; **Scenario 4**: severe pneumonia with ECG signs of acute RV strain. Kaplan–Meir survival curves related to the four scenarios in the lower part.

To identify predictors of all-cause mortality, univariate and multivariable Cox regression analyses were performed. First, among various clinical, blood, chest CT, and ECG variables, we identified the independent determinants (Table 3). At multivariable analysis only age (HR 1.48, 95%CIs 1.03–2.13, *p* = 0.0311) and ECG signs of acute RV strain (HR 2.46, 95%CIs 1.36–4.45, *p* = 0.0028) resulted in being independently associated with all-cause mortality. Moreover, the log-likelihood ratio test showed that ECG signs of acute RV strain had incremental prognostic value over chest CT scan, age, and C-reactive protein, as shown by the BIC decrease (Table 4) and the ROC curve where the AUC was significantly increased (Figure 1). Finally, Kaplan–Meier survival curves showed an incremental value of ECG signs of acute RV strain as a predictor of short-term prognosis over chest CT scan alone (Figure 2).

## 4. Discussion

We investigated in this study the potential of chest CT scan combined with standard ECG at hospital admission in predicting the 30-day prognosis of patients with COVID-19 pneumonia. The novel finding of our study is the independent and additive prognostic role of ECG signs of acute RV strain at admission on top of chest CT, clinical, and laboratory variables. Notably, both chest CT and ECG can usefully stratify the short-term mortality risk: patients with ECG signs of acute RV strain (S1Q3T3 pattern and/or right bundle branch block) have a worse short-term prognosis than patients with normal ECG or other ECG abnormalities have, and patients with severe pneumonia have a worse prognosis than patients with mild pneumonia. But the worst short-term outcome is associated with the coexistence of ECG signs of acute RV strain and severe pneumonia at CT scan. Figure 2 summarizes the four possible scenarios of COVID-19 pneumonia at hospital admission. The first is characterized by mild pneumonia without ECG signs of RV strain and has a favorable short-term prognosis (as shown in the Kaplan–Meier curves). The second scenario features mild pneumonia with ECG signs of acute RV strain and has a worse prognosis. The third scenario is characterized by severe pneumonia but without ECG signs of acute RV strain and is associated with a better short-term prognosis than scenario 2. Finally, the fourth scenario features severe pneumonia with ECG signs of acute RV, and it has the worst short-term prognosis. These findings may have crucial clinical implications and important pathophysiological insights may be gained from them.

### 4.1. Pathophysiological Insights

Among the highly heterogeneous symptoms of COVID-19 pneumonia, a distinctive phenotype has emerged, the so-called “vascular phenotype” involving both lung parenchyma and vascular damage. Supporting it are previous autopsy studies showing a variable frequency of pulmonary endothelialitis, thrombosis, and angiogenesis leading to severe impairment of the pulmonary circulation [7]. The notion of a vascular phenotype gained reinforcement from the study of Grasselli et al. on ventilatory and laboratory data in mechanically ventilated patients [6]. Our study supports the existence of this phenotype and confirms its poor prognostic association. Almost one in six patients of our cohort can be interpreted as having the ”vascular” (parenchymal and vascular), independent of whether they were mechanically ventilated or not. In the study of Grasselli et al., the prevalence of the vascular phenotype (based only on mechanically ventilated COVID-19 pneumonia patients) was roughly one in four patients [6]. A recent, relatively large study highlighted that ECG signs of RV pressure overload (right bundle branch block and S1Q3T3 pattern) can be detected in one-third of critically ill COVID-19 patients [13]. A similar prevalence of RV enlargement, suggesting RV pressure overload, has been confirmed by other small echocardiographic studies [10,11,18]. Taken together, all these studies contribute to shifting the initial attention from left to right ventricle, suggesting that myocardial inflammation is not the main cardiovascular complication in this disease. 

### 4.2. Clinical Implications

The combination of CT and ECG information provides an early powerful prognostic stratification tool, which can be integrated with the other adverse indicators of pulmonary circulation involvement such as plasmatic concentration of troponin and D-Dimer (8,9). In the early studies on the pandemic, the blood coagulation aspects received particular attention, and the prognostic role of increased levels of D-dimer immediately emerged [9,19,20]. However, D-dimer is a non-specific biomarker, which is elevated also in situations of severe general inflammation, and it does not indicate whether thrombosis affects the pulmonary macro and microcirculation or the extra-pulmonary circulation. Troponin elevation may also be useful to identify myocardial damage but, once again, it is highly non-specific since the injury may be in the left ventricle or RV, or both [8,21,22,23,24].

The therapeutic implications of an early recognition of a “vascular phenotype” are highly important and warrant all possible attention right at the beginning of the hospital stay. Both CT scan and ECG are performed in the early phases of hospitalization and may represent an ideal, simple non-invasive tool to characterize the patient, stratify the risk, and guide clinical decision-making, e.g., whether to anticipate invasive mechanical ventilation in high-risk patients or initiate immediately full-dose heparin treatment [25]. 

Finally, our data show that patients admitted to ICU or mechanically ventilated in the early stages had a better probability of survival, but these patients were also significantly younger, confirming that age, as our multivariable model shows, is one of the best predictors of mortality in COVID-19. 

## 5. Limitations

This was a retrospective study carried out on patients admitted with pneumonia to emergency care in the early phase of the COVID-19 pandemic. In the vast majority of cases, pre-admission ECG was missing, with no possibility of distinguishing between pre-existing and new-onset abnormalities. This limitation was unavoidable in a context of emergency crisis and “protected” access to the emergency room. Our pathophysiological insights are derived exclusively from ECG data at admission. Echocardiographic data were not available in most patients since this examination was of poor quality in mechanically ventilated patients and of high risk for healthcare workers in the pandemic context [26]. Finally, brain natriuretic peptide and troponin levels were not systematically assessed at hospital admission in these patients. 

## 6. Conclusions

Combining chest CT scan and ECG data improves risk stratification in COVID-19 pneumonia through the identification of a distinctive phenotype with both parenchymal and vascular damage of the lung, the so-called vascular phenotype. Patients with severe pneumonia at chest CT scan and ECG signs of acute RV strain have a very poor short-term prognosis.

## Figures and Tables

**Table 1:** Demographic and clinical characteristics, blood tests at hospital admission, and medication during hospitalization.

	Overall (151)	Survivors (97)	Deceased (54)	*p*
**Male, no. (%)**	95 (63)	62 (64)	33 (61)	0.73
**Age, yrs (IQR)**	71 (62–76)	69 (58–75)	74 (65–79)	0.006
**Hypertension, no. (%)**	117 (77)	75 (77)	42 (78)	1.00
**Dyslipidemia, no. (%)**	27 (22)	20 (27)	7 (13)	0.18
**Cigarette smoking, no. (%)**	22 (18)	15 (19)	7 (13)	0.63
**Diabetes, no. (%)**	28 (19)	20 (21)	8 (15)	0.51
**History of atrial fibrillation/flutter, no. (%)**	24 (16)	13 (13)	11 (20)	0.35
**Cardiovascular disease, no. (%)**	16 (11)	10 (10)	6 (11)	1.00
**Previous heart disease, no. (%)**	14 (9)	9 (9)	5 (9)	1.00
**Previous coronary artery disease, no. (%)**	9 (6)	4 (4)	5 (9)	0.28
**Previous peripheral artery disease, no. (%)**	4 (3)	2 (2)	2 (4)	0.62
**Chronic kidney disease (eGFR <60 mL/min/m2), no. (%)**	36 (29)	19 (25)	17 (35)	0.31
**Chronic obstructive pulmonary disease, no. (%)**	30 (20)	21 (22)	9 (17)	0.53
**Active malignancy, no. (%)**	13 (9)	7 (7)	6 (11)	0.55
**Systolic blood pressure at hospital admission, mmHg (IQR)**	125 (100–150)	135 (100–150)	123 (100–145)	0.37
**Diastolic blood pressure at hospital admission, mmHg (IQR)**	75 (65–85)	75 (65–85)	75 (65–85)	0.45
**Oxygen saturation at hospital admission, % (IQR)**	90 (85–93)	90 (86–93)	89 (84–93)	0.48
**Vasopressor drugs at hospital admission, no (%)**	12 (7.9)	6 (6.2)	6 (11.1)	0.35
**Intensive care unit, no. (%)**	61 (40)	49 (51)	12 (22)	0.001
**Invasive ventilation, no. (%)**	54 (36)	44 (45)	10 (19)	0.001
**Non-invasive ventilation, no. (%)**	104 (69)	68 (70)	36 (67)	0.72
***Blood tests at hospital admission***				
**Hemoglobin, g/dL (IQR)**	13.6 (12.3–14.8)	13.5 (12.4–14.5)	14.1 (12.2–15.0)	0.59
**D-dimer, mg/L (IQR)**	1.06 (0.70–1.95)	1.00 (0.67–1.65)	1.48 (0.90–2.46)	0.008
**LDH, U/L (IQR)**	393 (305–507)	393 (395–535)	391 (301–492)	0.61
**WBC, x10^3^/μL (IQR)**	4.54 (3.52–6.12)	4.54 (3.54–6.09)	4.78 (3.40–6.29)	0.99
**CRP, mg/dL (IQR)**	10.6 (5.6–16.4)	10.0 (5.1–14.2)	13.1 (7.4–18.0)	0.012
***Medication during hospitalization***				
**Heparin, no. (%)**	95 (77)	61 (80)	34 (71)	0.28
**Hydroxychloroquine** **, no. (%)**	107 (86)	67 (88)	40 (83)	0.59
**Antiviral drug, no. (%)**	103 (83)	64 (84)	39 (81)	0.81
**Tocilizumab** **, no. (%)**	7 (6)	5 (7)	2 (4)	0.71
**Steroids, no. (%)**	61 (40)	39 (40)	22 (41)	0.71

**Table 2:** Computed tomography scan and electrocardiographic characteristics.

	Overall (151)	Survivors (97)	Deceased (54)	*p*
***Computed tomography scan***				
**Pneumonia severity, no. (%)**				0.13
**Mild**	46 (31)	35 (36)	11 (20)	
**Moderate**	80 (53)	47 (48)	33 (61)	
**Severe**	25 (17)	15 (16)	10 (19)	
**Moderate/severe pneumonia, no. (%)**	105 (70)	62 (64)	43 (80)	0.044
***Electrocardiogram***				
**Abnormal electrocardiogram, no. (%):**	121 (80)	74 (76)	47 (87)	0.13
**Heart rate, bpm (IQR)**	82 (70–97)	80 (68–95)	85 (73–100)	0.23
**Atrial fibrillation/flutter, no. (%)**	15 (10)	6 (6%)	9 (17%)	0.039
**QRS interval, ms (SD)**	90 (80–100)	90 (80–98)	90 (80–110)	0.47
**Low QRS voltage peripheral leads, no. (%)**	4 (2.8)	4 (4.3)	0 (0.0)	0.30
**Pathologic Q waves, no. (%):**	10 (7)	4 (4.1)	6 (11.1)	0.17
**Left ventricular hypertrophy, no (%)**	9 (6)	7 (7.2)	2 (3.7)	
**S1Q3T3 pattern, no. (%)**	12 (8)	5 (5)	7 (13)	0.089
**RBBB, no. (** **%)**	25 (17)	9 (9)	16 (30)	0.001
**RV strain, no. (%)**	29 (19)	10 (10)	19 (35)	<0.001
**Left anterior hemiblock, no. (%)**	9 (6)	4 (4)	5 (9)	0.20
**LBBB, no (%)**	4 (3)	4 (4)	0 (0)	0.13
**Non-specific RA, no. (%)**	33 (23)	19 (21)	14 (27)	0.41
**QTc, ms (IQR)**	445 (414–464)	443 (415–460)	448 (413–470)	0.36

**Table 3:** Univariate and multivariable Cox regression analyses.

	Univariable			Multivariable Backward Elimination (FULL MODEL)		
	HR	95ci	*p*	HR	95ci	*p*
**Sex**	0.908	0.525–1.569	0.729			
**Age**	1.647	1.139–2.381	0.008	1.484	1.032–2.133	0.0311
**LDH**	1.061	0.728–1.546	0.758			
**WBC**	1.125	0.902–1.405	0.296			
**CRP**	1.359	0.907–2.036	0.137	1.46	0.96–2.219	0.0766
**D-dimer peak**	1.335	1.051–1.697	0.018			
**Oxygen saturation**	0.83	0.595–1.158	0.273			
**Non-severe pneumonia**	1.956	0.988–3.872	0.054			
**Severe pneumonia**	2.596	1.075–6.266	0.034	2.043	0.835–5	0.12
**ECG signs of acute RV strain**	3.094	1.762–5.431	<0.001	2.464	1.364–4.451	0.0028
**Comorbidity**	0.977	0.788–1.211	0.829			

Comorbidity: presence of at least one cardiovascular risk factors between hypertension, dyslipidemia, cigarette smoking, diabetes, history of atrial fibrillation/flutter, cardiovascular disease, previous heart disease; CRP: C-reactive protein; ECG: electrocardiographic; LDH: lactate dehydrogenase; RV: right ventricular; WBC: white blood cell.

**Table 4:** Prognostic value over chest CT scan.

	Loglik	Chisq	*p*(>|Chi|)	BIC
**Age + CRP + pneumonia severity + RV strain**	−246.767			517.458
**Age + CRP + pneumonia severity**	−250.835	8.166	0.004	521.615

CRP: C-reactive protein; BIC: Bayesian information criterion.

## Data Availability

Data was registered on ClinicalTrials.gov (identifier: NCT04367129).

## Abbreviations

AUC	area under the curve
BIC	Bayesian information criterion
COVID-19	coronavirus disease-2019
CT	computed tomography
ECG	electrocardiogram/electrocardiographic
HR	hazard ratio
IQR	interquartile range
RBBB	right bundle branch block
RV	right ventricular
